# Microwave-Assisted Hydrodistillation of Hop (*Humulus lupulus* L.) Terpenes: A Pilot-Scale Study

**DOI:** 10.3390/foods10112726

**Published:** 2021-11-07

**Authors:** Lorenzo Lamberti, Giorgio Grillo, Lorenzo Gallina, Diego Carnaroglio, Farid Chemat, Giancarlo Cravotto

**Affiliations:** 1 Dipartimento di Scienza e Tecnologia del Farmaco, University of Turin, Via P. Giuria 9, 10125 Turin, Italy; lorenzo.lamberti@unito.it (L.L.); giorgio.grillo@unito.it (G.G.); lorenzo.gallina@edu.unito.it (L.G.); 2 Baladin S.S. Agricola, Via Carrù 23, 12060 Piozzo, Italy; 3 Milestone Srl, Via Fatebenefratelli, 1-5, 24010 Sorisole, Italy; d.carnaroglio@milestonesrl.com; 4 GREEN Extraction Team, INRAE, UMR 408, Avignon University, 84000 Avignon, France; farid.chemat@univ-avignon.fr

**Keywords:** green extraction, hops (*Humulus lupulus* L.), microwave-assisted hydrodistillation, terpenes, pilot-scale extraction

## Abstract

Interest in essential oils has consistently increased in recent years. Essential oils have a large variety of applications in multiple fields, including in the food, cosmetics and pharmaceutical industries. The volatile fraction (VF) in hops (*Humulus lupulus* L.) fits within this domain as it is primarily used in the brewery industry for the aromatization of beer, and is responsible for the floral and fruity tones. This work aims to design an optimized extraction protocol of the VF from hops, using microwaves. Microwave-assisted hydrodistillation (MAHD) has been developed to reduce energy and time consumption in lab-scale reactors up to industrial-scale systems. Hops are principally available in three forms, according to a brewery’s applications: (i) fresh (FH); (ii) dried (DH) and (iii) pelletized (PH). In this work, all three forms have therefore been studied and the recovered volatiles characterized by means of GC-MS. The optimized lab-scale MAHD protocol gave the best extraction yield of 20.5 mL_VF_/kg_dry matrix_ for FH. This value underwent a slight contraction when working at the highest matrix amount (3 kg), with 17.3 mL_VF_/kg_dry matrix_ being achieved. Further tests were then performed in a pilot reactor that is able to process 30 kg of material. In this case, high yield increases were observed for PH and DH; quadruple and double the lab-scale yields, respectively. In addition, this industrial-scale system also provided marked energy savings, practically halving the absorbed kJ/mL_VF_.

## 1. Introduction

Hops are the inflorescence produced by *Humulus lupulus* L., which belongs to the Cannabaceae family. The plant requires temperate climates and is native to Europe, southwestern Asia and North America [1].

The average production of hops fluctuates around 100 thousand tons per year. The main producers are the United States, Germany and the Czech Republic, with respectively, 44.3, 39.0 and 6.1 thousand tons [2]. Furthermore, 90% of the world production of hop inflorescences is destined for the brewing industry [3], where only the female plant flowers are exploited for their aroma and the typical bitterness that is associated with the beer. All of the flavoring molecules are found in the lupulin glands [4], which can be found under the bracts near the center of the flower. The composition of these compounds depends on the hop variety and plays a crucial role in the final fragrance.

There are several classes of compounds that are responsible for the organoleptic features of beer. They can be divided into resins and essential oil (EO). In detail, there are two types of resins: (i) hard resins, insoluble in hexane, approximately 5% of the total resin content; and (ii) soft resins, soluble in hexane. The latter are divided into α and β acids. The α-acids are primarily responsible for the bitter hop taste in beer [5].

Conversely, EOs do not affect the bitterness, but are responsible for the aroma. They bring citrus-like, floral, fruity notes, spicy, woody, herbal and grass aroma fragrances, depending on the variety of the hops used and their cultivation environment [6,7]. The EO produced by *Humulus lupulus* L. can be divided into three subgroups: hydrocarbons, oxygen-bearing components and sulfur-containing molecules.

Hydrocarbons account for approximately 75% of the entire EO, and can be divided into monoterpenes and sesquiterpenes [8]. The four key compounds are myrcene (monoterpene), caryophyllene, farnesene and humulene (sesquiterpenes) [9]. Due to the strong influence that they have on beer characteristics, the ratio between myrcene and humulene is generally used to categorize hop batches (noble hops generally show a humulene/myrcene ratio that exceeds 3.5). It is important to note that the EO can also deteriorate over time (as can the soft resins) due to oxidation; during the pelletization process for example. For this reason, there are apparent aroma differences between fresh and pelletized versions of the same hop batch [10,11]. Oxidized molecules are usually considered because of the grassy touch that they provide to beers, as an organoleptic property.

Oxygen-bearing components make up approximately 25% of the hop essential oil [8]. They cause the floral and herbal aroma that characterizes many hops, such as the Cascade and Continental [12]. Of these components, linalool and geraniol are worthy of particular mention.

Sulfur-containing components are the last subgroup of EO molecules. An excess of sulfury tones is generally defective for beer. It is known that malt and the fermentation process are the major sources of sulfur flavors, but hops can also contribute [10].

In addition to its flavoring features, the components of hop EO can be exploited for a wide range of purposes, including therapeutic, cosmetic and nutritional applications [13,14]. Furthermore, particular interest has been shown in their several biological properties; they demonstrate antioxidant, antimicrobial and antiviral attributes, [9,14,15], as well as antitumoral effects [16,17] and uses in pest control [18].

The profile of the volatile fraction of hops mainly depends on genetic and environmental factors, together with the extraction protocol applied. The traditional method suggested by ASBC (American Society of Brewing Chemists) consists of steam distillation that can last up to 7 hours. This method is highly time and energy consuming and is only applied to analyze hop quality [19]. The conventional methods generally applied to recover the EO are steam distillation, hydrodistillation, maceration and absorption [20,21].

Currently, the conventional extractions used to recover natural products generally include several unitary operations, including plant pretreatment (drying, comminution, etc.), material extraction itself and finally downstream processes (separation, evaporation, etc.). All of these steps are generally time and energy consuming when not optimized, but the extraction stage, in particular, can involve either considerable water, solvent or both, consumption, and generate a large amount of waste materials [22].

In recent years, with the increasing concern for environmental issues, companies are adapting their ethical values and productive practices. The pursuit of global sustainability and “green industry” requires real solutions that can minimize environmental impact, while maintaining product quality [23,24]. In this context, process intensification is imperative; enhancing extraction efficiency, producing safer extracts of higher quality and reducing the unitary operations. These expectations rely on enabling technologies, such as microwave (MW), ultrasound, and super- and sub-critical fluid extractions [25,26,27,28], together with the application of new green solvents [29,30,31]. This type of approach, which has emerged over the last decade, has now suitably evolved towards up-scaling [32,33,34,35,36]. Protocols that have been optimized on the lab-scale require pilot and pre-industrial prototypes, which are a crucial step to bridge the gap between academia and industry, and can consistently pave the way towards process sustainability.

Several works have reported the application of supercritical CO_2_ (sc-CO_2_) as part of transposing the awareness of ecological and environmental issues to the recovery of the hop volatile fraction. This technique exploits the peculiar polarity and viscosity that CO_2_ achieves in the supercritical state. In this form, it behaves similarly to ethanol and methanol, extracting both α-acids and the lipophilic fraction (also volatiles) [37,38,39]. The extract obtained using this technique is stable for long periods and can be introduced directly into the brewing process as CO_2_ is desired in the final product. Nevertheless, the recovered product represents a mix of several classes of compounds and is not purely composed of volatiles.

In an attempt to selectively recover hop terpenoids, this study focuses on MW-assisted hydrodistillation (MAHD). MAHD is an emerging technology in the extraction and purification of the volatile fractions of plants, and is far more efficient than classic hydrodistillation [40,41]. To the best of our knowledge, this technique has not yet been applied to *Humulus lupulus* L., whilst only one study has reported the use of a sc-CO_2_ hop extract as a starting material [42]. MAHD is an efficient alternative for the recovery of hop terpenes, and can provide reduced process times and energy consumption. Furthermore, the biomass is treated more homogeneously as the irradiation is not focused at the bottom of the reactor, as occurs in classical distillation [43,44]. The reduction in extraction time and uniform nature of the heating are also crucial for the quality of the volatiles recovered, as they could possibly undergo oxidation during the process.

## 2. Materials and Methods

### 2.1. Materials and Vegetal Matrix

Chloroform (ACS grade, ≥99%) and the analytical standards (myrcene, caryophyllene, farnesene, humulene) used for GC-MS analysis were purchased from Sigma-Aldrich (St. Louis, MO, USA).

Hops, used for the hydrodistillation protocols, belong to the cascade variety, and were harvested at the end of August 2020 in the Piedmont region (Italy). After harvesting, the cones were dried for 12 hours at 42 °C, decreasing the average humidity to 8%. The material was left for 24 hours at room temperature before pelletization. Dried cones were left to rest for 24 hours and their humidity rose to 10/11%, and the hops then underwent pelletization. This process started with a fine mincing of the cones and the collected powder was then pressed through an extruder to create the pellets. The temperature increased substantially during pelletization and reached 50 °C. Afterwards, the pellets produced were small cylinders of 1/2 cm in length and 0.5 cm in diameter. The final humidity reached by hop pellets at the end of the process was 12%. The materials were identified as fresh hops (FH), dried hops (DH) and pellet hops (PH) in accordance with the different post-harvesting processes. All of the biomasses were stored under vacuum at −18 °C for their preservation. The water content was evaluated via thermogravimetric analysis by leaving an average weight of 1.5 g of biomass at 100 °C overnight and measuring the relative weight loss. The test was performed in triplicate and the results are reported in Table 1.

### 2.2. Microwave-Assisted Hydrodistillation (MAHD)

**Figure 1:**
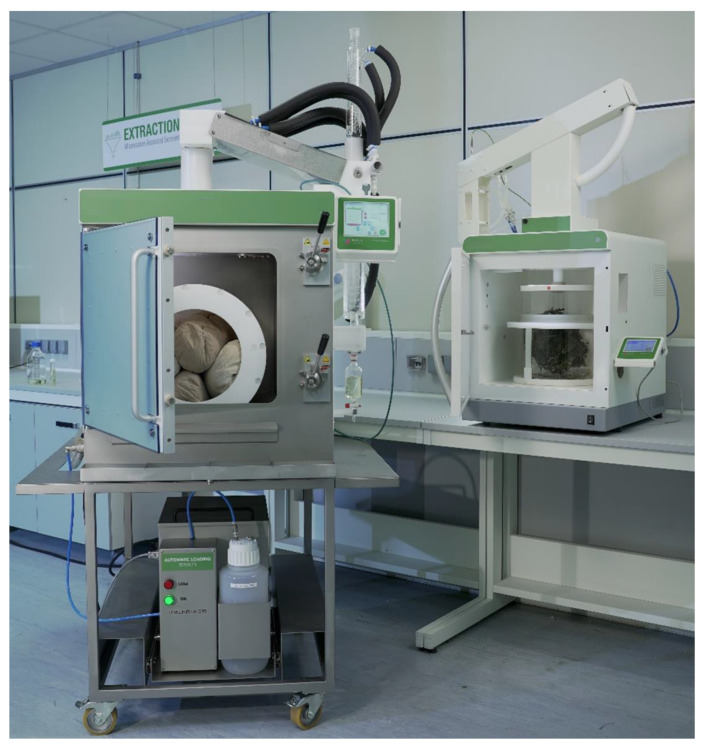
ETHOS X (right) and an ETHOS XL (left) extractors (Milestone srl, Bergamo, Italy).

MAHD was carried out using ETHOS X and ETHOS XL extractors (Milestone srl, Bergamo, Italy, see Figure 1). Extractions were performed in triplicate and expressed as average ± SD. For the sake of clarity, all the runs reported are numbered progressively across the manuscript.

#### 2.2.1. Standard Operating Procedure (SOP) and Soaking Optimization

The first tests were performed using ETHOS X, with a 5 L vessel, with a standard operating procedure (SOP). The SOP is made up of a moistening phase and a standardized MAHD protocol, with fixed MW-irradiation power and time as reported in the supporting material (Table S1). In the SOP, the water/cone ratio of the moistening pre-treatment depends on the matrix type; 0.5 L/kg for fresh biomass and 1 L/kg for dried biomass (DH and PH). In addition, this study evaluated different moistening ratios for fresh hops; 0.25, 1 and 2 L/kg. The vegetal material (up to 1200 g) was soaked directly in the 5 L ETHOS X vessel for 15 minutes with mild manual agitation. The extraction procedure required the presence of at least ¼ empty space in the vessel.

#### 2.2.2. Mild Irradiation MAHD Protocol

The effect of milder MW-irradiation on the hop volatile-fraction yields and quality was investigated by means of a dedicated MAHD protocol, which we had previously optimized on sensitive biomass (*Cannabis sativa* L.) [41]. The hydrodistillation conditions are summarized in Table 2.

#### 2.2.3. Fresh-Hop MAHD: ETHOS X 12 L Scale-Up

As MAHD had the best performance on fresh hops, the extraction scale-up of this matrix was investigated. In detail, fresh-cone cascades were processed in a 12 L vessel, which allowed approximately 3 kg of biomass to be treated. The MAHD protocol reported in Section 2.2.2 was applied, with the last step (Table 2, step 4) being extended, up to 6 hours, to increase sample collection.

#### 2.2.4. MAHD: ETHOS XL

A new reactor (ETHOS XL, Milestone srl, Bergamo, Italy, Figure 1) was tested, for the first time, to further investigate the feasibility of pilot MAHD.

The ETHOS XL presents itself as a cube with 0.5 m sides. The bottom of the reactor has a depression that is filled with water during processing. A 45 L rotating drum, which is capable of maintaining a homogenized moistening level and the homogenized diffusion of microwaves over the treated biomass, is located in the center of the reactor. The magnetron in the extractor has 4 kW of power, although this is not the only heating source provided by the ETHOS XL. Each internal panel has a resistance of 1.8 kW and these maintain constant temperature inside, and enhance microwave activity in the biomass. The system has a recycling loop that prevents the reduction of the overall moistening level. The vapors released during the extraction are collected from the top of the reactor and sent to three condensers that are located in parallel and condense the vapors released. The extracted essential oil is stored in a chilled tube in which the separation with the aqueous solvent takes place. To ensure that no volatile compounds are lost, a fourth condenser is placed on top of the separating tube. The chiller that is paired with the ETHOS XL requires at least 5 kW of power to ensure good refrigeration across all of the condensers. The reactor is equipped with a thermocouple in the vapor-collecting region, which enables direct temperature control to be exerted via variable power output. Furthermore, a 9 L water pool is present inside the cavity to avoid combustion phenomena and strongly decreases the energy consumption. This type of set-up allowed a reduced water/cone ratio (0.5 L/kg, as SOP reports) to be used. The reactor was run at both half (low loading, LL) and full (full loading, FL) capacity to test pilot performance. In the first case, the water pool required only a 5 L charge.

Due to the cavity pre-heating system and magnetron-power variation, a dedicated irradiation protocol was tested, while step duration was modified according to reactor loading. Conditions are summarized in Table S2. 

The protocols were used on fresh, dried and pelletized hops.

#### 2.2.5. MAHD Vacuum

**Figure 2:**
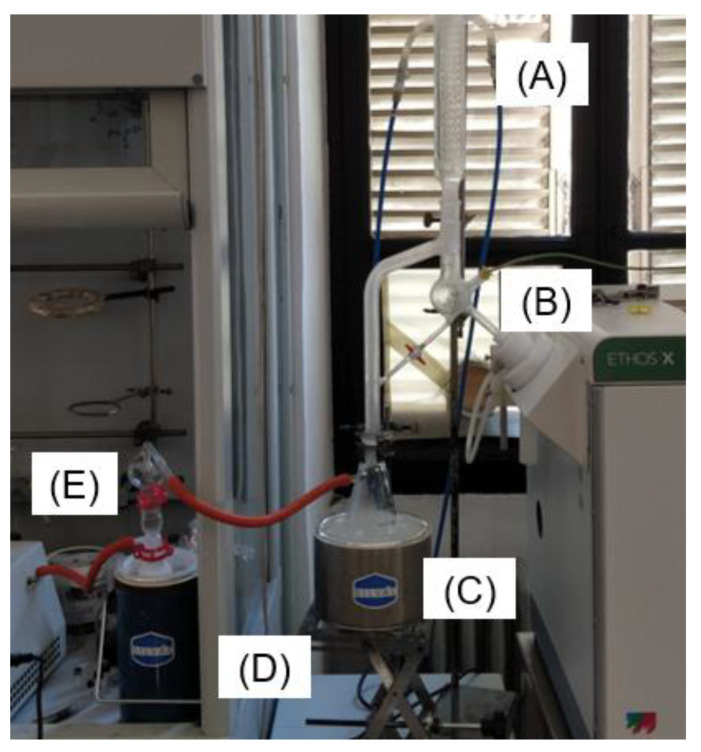
ETHOS X vacuum set-up; (A) condenser, (B) thermocouple, (C) collecting flask, (D) liquid-nitrogen trap, (E) pump.

In order to accelerate the distillation process, a different approach that used a customized ETHOS X set-up was investigated. Suitable glassware facilitated the incorporation of a vacuum pump into the system, while the vapor-collecting region was also equipped with a thermocouple. Similarly, to ETHOS XL, this setup enabled direct temperature control to be performed with automatic adjustments to power output being made by the system. Lastly, an additional liquid-nitrogen trap was added to avoid losses of the volatile fraction (see Figure 2). In detail, 700 g of fresh cascade hops were loaded with 700 mL of water into a 2 L vessel. Two different extraction protocols were applied: a mild one that reached 95 °C with a maximum 1200 W power being delivered (protocol A); and a harsher one that reached 99 °C with a maximum 1600 W power being delivered (protocol B). Both protocols are reported in the supporting material (Table S3).

Due to the high evaporation rates that occurred during processing, 400 mL of water were introduced into the vessel in 4 steps. Every time 100 mL was sampled, the extraction was stopped and an equal volume of fresh distilled water was introduced slowly into the vessel.

### 2.3. GC-MS Analysis

The GC-MS qualitative analyses of the volatile fractions obtained using MAHD were performed in an Agilent Technologies 6850 Network GC System fitted with a 5973 Network Mass Selective Detector, a 7683B Automatic Sampler and a capillary column Mega 5MS (length 30 m; i.d. 0.25 mm; film thickness 0.25 μm, Mega s.r.l., Legnano, Italy). Each sample was prepared by mixing 5 μL of the volatile fraction with 1 mL of chloroform, with this being repeated in triplicate. The adopted chromatographic protocol is reported in the supporting material (Table S4). The identification of the individual compounds was performed using two approaches: (1) by comparing the retention time and mass spectrum with standard compounds, and (2) using the GC-MS Wiley275 and NIST05 GC libraries from the acquired chromatograms, and only considering matching levels of over 95%. The summed areas of the relevant peaks were normalized to 100%. Relative peak areas, calculated as percentages, were used to evaluate extract composition.

## 3. Results and Discussion

### 3.1. MAHD

The MAHD extraction tests and their respective extraction yields are summarized in the Supplementary Material (Table S5 and Figure S1).

#### 3.1.1. SOP

The SOP was applied to evaluate the suitability of different types of biomasses for the recovery of the volatile fraction. As reported in Table 3, dry hops appear to be the best-performing matrix (9.25 mL_VE_/kg_dry matrix_ of DH vs. 5.00 and 3.30 mL_VE_/kg_dry matrix_ of FH and PH, respectively). Nevertheless, normalization that was performed on biomass water content shows that working with fresh material led to better volatile-fraction recovery (16.67 mL_VE_/kg_dry matrix_ of FH vs. 10.51 and 3.75 mL_VE_/kg_dry matrix_ of DH and PH, respectively). 

Accordingly, the influence of the fresh-hop moistening process was evaluated. Thus, it was possible to evaluate even limited recovery variations. In detail, this study evaluated soaking ratios of 0.25, 1 and 2 L/kg. As reported in Table 4, it is necessary to use a ratio of at least 0.5 L/kg for fresh biomass. Lower amounts of water (0.25 L/kg) triggered combustion phenomena, leading to the unsuccessful recovery of the volatile fraction. Conversely, higher additions of water (1 L/kg) did not appear to modify the extraction yield, with there being a little production decline for 2 L/kg (16.67 mL_VE_/kg_dry matrix_ vs. 15.28 mL_VE_/kg_dry matrix_, entries 5 and 6, respectively).

#### 3.1.2. Mild Irradiation MAHD

According to our previous experience on volatile-fraction recovery by means of MW irradiation, the three different hop typologies were tested by applying mild MAHD (see Table 5). 

This protocol is able to save the lighter volatile fraction by avoiding condensation problems and leakages due to overheating. At the same time, this approach reduces the formation of the hot-spots that lead to biomass degradation and interfere with the final flavor of the recovered product, due to degradation and compound alteration.

**Figure 3:**
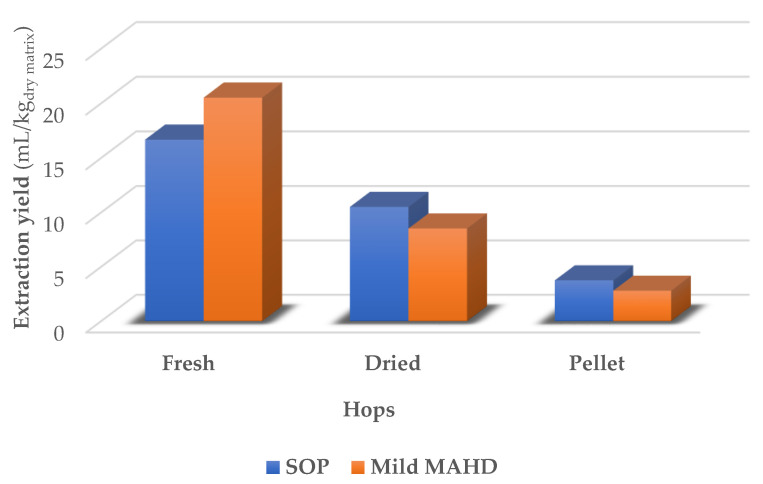
Volatile-fraction yield according to MAHD protocols.

As reported in Figure 3, mild MAHD provided better performance on the fresh hops, reaching an approximate 23% rise compared to SOP, with the fresh hops again being confirmed as the most promising matrix. Conversely, dried hops and pellets suffered average yield decreases of 18% and 25%, respectively. This trend supports the abundance of extremely volatile compounds that are present in recently harvested hops, and the fact that these molecules are partially lost with excessively harsh extraction protocols. Dry biomass, such as dried cones and pellets, on the other hand, have already been depleted of this light fraction during post-harvesting treatment. The L/S was maintained at 1 L/kg as in the reference protocol [41], as it has already been demonstrated in Section 3.1.2 that moistening does not significantly affect the yield. In summary, mild MAHD appears suitable for FH extraction, while slight yield contractions are observed for DH and PH. 

#### 3.1.3. ETHOS X 12 L Scale-Up: Fresh Hops

Interest toward volatile-fraction production and exploitation has driven the development of systems suitable for industrial-scale production. In our work, the very first step was taken by investigating the effects of scale-up on MAHD yield and by simply employing a larger extraction vessel. For this purpose, the starting load was tripled and tested in the same MW device.

**Figure 4:**
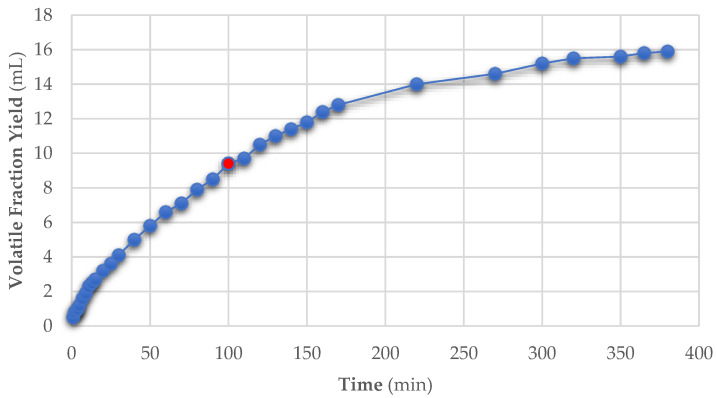
Extraction timeline, the red dot shows the end of the small-scale protocol.

To better understand process evolution, the volatile-extraction rate was monitored on FH applying mild MAHD protocol (Figure 4).

At the end of the mild MAHD protocol, only 9.4 mL of product was extracted (18.5 mL expected), although linear output was maintained. Thus, the process was continued until a plateau was reached with the hydrodistillation period being extended to 360 min. At the end, 15.9 mL of volatiles were collected (Table 5, entry 10), with a moderate loss of 16% compared to the small-scale expected yield, and an overall yield of 17.67 mL_VE_/kg_dry matrix_ was achieved. From a physical point of view, the biomass was mostly similar to previous samples at the end of the six hours of hydrodistillation, except for a small combusted area near the vessel’s surface. From this detail, it is possible to assume that higher biomass loadings require more homogeneous irradiation inside the reactor chamber. Nevertheless, it is still remarkable that only a negligible fraction suffered from this effect. This test proved that it is possible to treat higher biomass quantities using a larger vessel, although the time required to reach comparable extraction yields increases proportionally. Our investigation highlighted the possibility to process more biomass per extraction batch though some adjustments to the extractor set up are required.

### 3.2. Pilot MAHD: ETHOS XL 

According to the results and observations gathered during the experimental set-up and extraction screenings, the main issue for pilot-scale hydrodistillation is poor mass transfer. Large quantities of material interfere with vapor-flow dynamics because of the strong packing, which is then worsened by biomass texture. Fresh hops, due to their coarse size and heterogeneous composition, show better behavior, whereas dried cones and pellets suffer considerably from this effect. For pellets, in particular, this phenomenon is visually embodied by a swelling of approximately 15–20% (with respect to the loading volume) during the process, due to trapped vapors. Thus, the higher yield for fresh hops is not explained solely by the higher concentration of volatiles, which are lost in the drying process for dried and pelletized cones. In common production practices, breweries usually exploit hops in the pellet form, making this format the most available and attractive for pilot-scale applications, despite being the most troublesome.

A pilot reactor has been developed specifically to solve the scalability issues by focusing on enhancing mass transfer, which is a bottle-neck in hydrodistillation process scale-up. Moreover, the MW-distillation apparatus was merged with a hybrid technology, with the aim of reducing energy consumption. 

The screening approach used to investigate pilot performance involved an initial set of extractions for all three typologies of biomass, with adjustments being made to the lab-scale reactor loadings (2–3 kg, LL, Table 6, entries 11–13).

The introduction of a mobile body provided a mass-transfer enhancement as well as homogeneous wetting and exposure to MW. A direct effect can be observed on the dry cones and pellets, with the average mL_VE_/kg_dry matrix_ yield increasing (8.52 vs. 11.76 and 2.80 vs. 6.25, respectively, entry 8 vs. entry 12 and entry 9 vs. entry 13). Fresh hops maintained comparable productivity.

**Figure 5:**
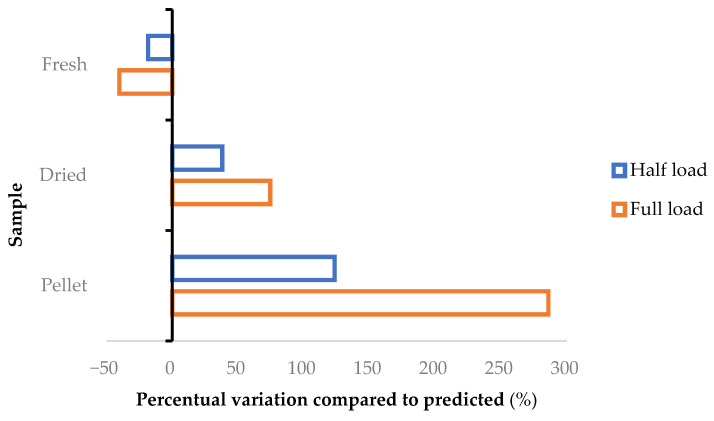
Percentage variation in mL of volatile fraction, according to the small-scale projections.

Finally, the irradiation chamber was tested for a FL (Table 6, entries 14–16), whose weight was strictly related to the type of biomass. In detail, volatiles yield for FH are roughly unchanged, while a small increase can be observed for DH, reaching a dramatic enhancement for PH, almost doubling the process outcome. In Figure 5 we can see how the pilot prototype was able to enhance the hydrodistillation yields for dried and pelletized hops, compared to the small-scale projections. 

In particular, pellet productivity was approximately quadrupled, reaching 10.80 mL_VE_/kg_dry matrix_ (entry 16), from the 2.80 mL_VE_/kg_dry matrix_ (entry 9) obtained from the small-scale process. Similarly, dry cones nearly doubled the overall outcome, increasing from 8.52 (entry 8) to 14.87 mL_VE_/kg_dry matrix_ (entry 15).

Fresh biomass, on the other hand, suffered slight decreases with process scale. This effect can be explained by the soaking/charge step, whose relevance rises proportionally with matrix load. Fresh hops are very attractive thanks to the high amount of light volatiles, which are lost during the desiccation and pelletization protocols. Extending early-stage protocols (such as soaking, mixing and loading) contributes to wasting this fraction. Nevertheless, it is unreasonable to process huge volumes of newly harvested plants without expensive measures (cold chains, either inert atmospheres, vacuum systems or in combination) precisely because of their instability (in addition to fermentation and degradation). The strategic choice of breweries to work with pelletized hops, which can provide a constant and stable supply, is a practical example. Thus, it is possible to consider that large-scale extraction for industrial purposes will focus on pellets and dried cones, as they are simply available for storage. Smaller applications (still approximately 3 kg) can be envisaged for high quality processes using fresh biomass with the aim of recovering the most volatile compounds.

### 3.3. MAHD: Vacuum Set-Up

**Figure 6:**
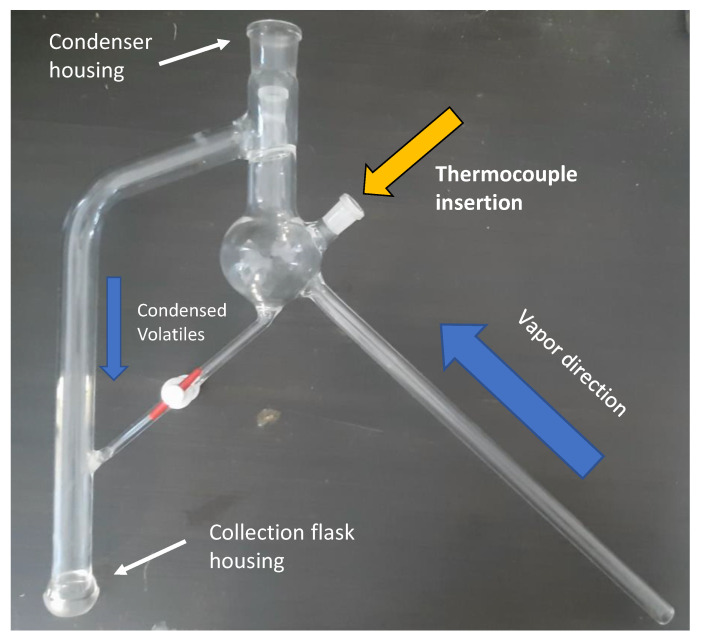
Customized glass apparatus implemented during MAHD under vacuum.

This particular set-up was customized in order to find a reliable approach to switching from the typical power-control to a temperature-control system. These systems are able to detect the temperature of the extraction vessel using either IR probes or optical fibers. Nevertheless, these two approaches suffer from a certain degree of uncertainty; IR can only properly detect superficial temperatures, meaning that increasing vessel dimensions inevitably leads to large deviations, whereas optical fibers are difficult to insert inside the system due to sealing issues. Moreover, speaking of a static device, the detected T may be not representative of the whole vessel. For these reasons, the most common MAHD devices are set to power-control, without a PID associated with temperature evolution. The proposed vacuum set-up was tested in order to verify the possibility of adjusting power irradiation according to vapor temperature. It is possible to observe the detail of the dedicated glass apparatus in Figure 6 (also visible in Figure 2). 

The optical fiber is placed right at the vapor output, whilst a slight vacuum (approximately 0.05 MPa) is applied to enhance the distillation, allowing higher extraction yields to be reached with lower energy consumption. The MAHD were tested at two different temperatures, namely, 95 and 99 °C (Table 7), to screen the flexibility of the system. From an engineering point of view, the heating profile and irradiation trend accurately followed the set parameters. The wattage emissions were strongly reduced, which decreased the energy consumption accordingly. For example, in the ramp stage, mild MAHD absorbed 1245 W. Hence, vacuum protocols saved approximately 40% and 20.6%, at 95 and 99 °C, respectively (entries 17 and 18). 

Unfortunately, the volatile fraction recovered by means of the vacuum set-up was found to be smaller than that of SOP MAHD (16.67 mL_VF_/kg_dry matrix_). An average reduction of approximately 45% was observed, with a minimum gap of 4% when comparing the 95–99 °C protocols. These results can be explained if we assume that the performance of the trap section, which serves to avoid losses due to pump suction (Figure 2, elements C,E), was inefficient. Furthermore, the tests were performed on the most characterized matrix, namely, the fresh hops, which is, on the other hand, the richest in extremely volatile compounds, and presumably the most difficult to trap. On the basis of these considerations, future evaluations would involve a more sophisticated cooling system before the pump, such as a vertical condenser with a collecting flask that is not directly connected to the pump. Since additional customization is required, further investigations with this set-up are suspended for the moment. Nevertheless, a promising approach to introduce temperature control as an instrumental parameter during MAHD is reported herein.

### 3.4. MAHD Energy Consumption

Process energy consumption is a critical factor that must be evaluated for suitable scale-up design. We therefore monitored the average Watts absorbed by the ETHOS X and ETHOS XL. Furthermore, chillers were taken into account due to their significant impact on total energy costs. From this data, it was possible to calculate the overall Joules used over the entire process.

It is important to remember that the applied protocols treated different quantities of matrix and produced different amounts of essential oil. For this reason, extraction energy consumption was normalized to the volumes of volatiles collected.

**Figure 7:**
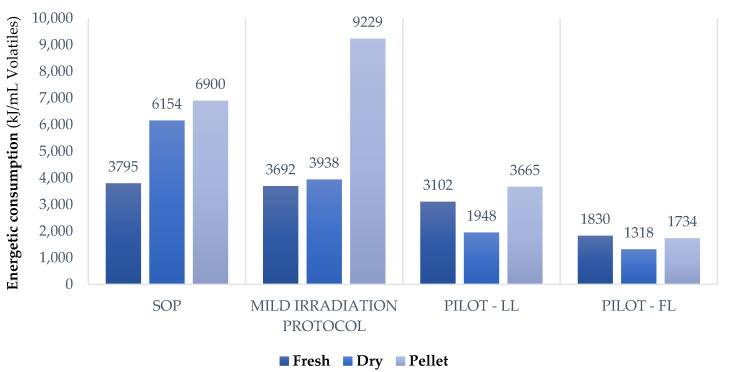
Energy consumption comparison. Chiller systems are computed.

As shown in Figure 7 (Table S6), FH submitted to the mild irradiation protocol is characterized by the lowest energy consumption when using the ETHOS X. All the other extractions showed higher consumption when carried out on the same system, especially when using pellets, due to the lower extraction rates. Conversely, the energy demands of the pilot reactor appear to be far better than those achieved using the lab-scale device, in particular when the instrument was used at full capacity (pilot-FL). This trend proves the effectiveness of the MW hybrid approach, together with the design of appropriate mixing/soaking apparatus.

### 3.5. Volatile Fraction Evaluation

During the GC-MS analysis, we focused our attention on the four characteristic hop compounds: myrcene, caryophyllene, farnesene and humulene. These terpenoids can be categorized by their chemical structure; monoterpenes (myrcene) and sesquiterpenes (caryophyllene, farnesene, humulene). The components of this last group have higher molecular weight and boiling points. As expected, myrcene is the most abundant terpenoid recovered from hops, followed by humulene, caryophyllene and farnesene. This trend was observed independently of matrix nature (FH, DH, PH). This is in line with the literature data on the cascade variety of hops [12]. An overview of this component distribution across the screened samples is reported in the Supplementary Material (Figure S2).

#### 3.5.1. SOP

**Figure 8:**
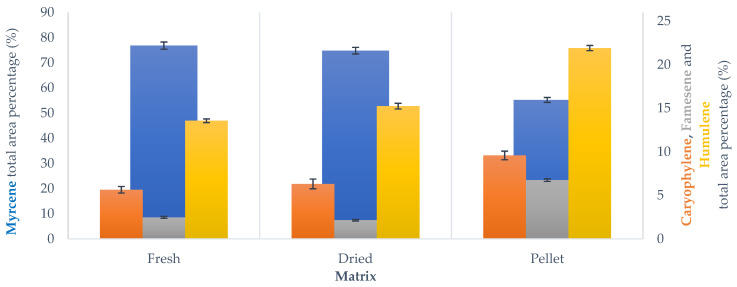
Main terpenoid distribution across SOP MAHD screenings, GC-MS analysis. Results reported as average ± SD.

In the extraction carried out using SOP, it is possible to notice a high similarity between fresh- and dried-hop volatile fractions. (Figure 8). On the other hand, PH are characterized by a higher sesquiterpene-fraction concentration than the other matrices. This difference is probably caused by the pelletization process, as the mincing and extrusion steps occur at high temperature. Although it is considered to be a fast procedure, it can affect the terpenoid compounds. Since myrcene is the most volatile component, it is particularly prone to heat-related loss. It is possible to reduce pelletization temperature using a refrigerated system in order to lessen this phenomenon. For example, several industries exploit a liquid nitrogen system during mincing and extrusion. As these two steps are the crucial points, using a coolant afterwards would not be effective.

#### 3.5.2. Mild Irradiation MAHD and Scale-Up

**Figure 9:**
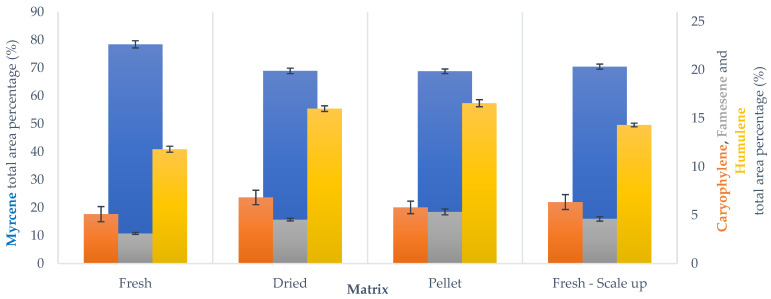
Main terpenoid distribution across mild irradiation MAHD and scale-up screenings, GC-MS analysis. Results reported as average ± SD.

Much higher resemblance between the different biomasses was observed in the analysis of the volatile fractions recovered using the mild irradiation protocol, as can be appreciated in Figure 9. However, DH and PH still have a higher concentration of sesquiterpenes and a lower one of myrcene. 

The scale-up test that was carried out using the 12 L vessel (Table 5, entry 10) showed no considerable differences in sesquiterpene ratios, compared to the small-scale experiment, and a contraction in myrcene yield (approximately 8%). This reduction reflects the mild decrease in overall dry yield (17.67 mL_VE_/kg_dry matrix_ vs. 20.51 mL_VE_/kg_dry matrix_) and can be attributed to FH being very sensitive to the soaking/charge steps that are necessarily extended due to the higher amount of starting material. Nevertheless, this loss can be considered negligible when we consider the three-fold increase in productivity (up to 3 kg). Thus, it is reasonable to employ the larger vessel without appreciably affecting the composition of the volatile fraction collected.

#### 3.5.3. Pilot MAHD: ETHOS XL

**Figure 10:**
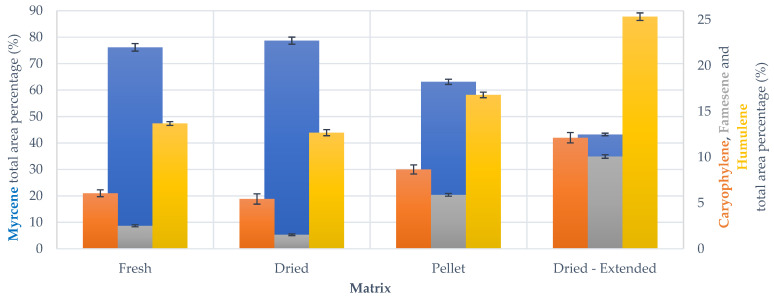
Main terpenoid distribution across ETHOS XL-MAHD (full load, FL), GC-MS analysis. Results reported as average ± SD.

The analysis of the volatiles recovered from the ETHOS XL confirmed negligible fluctuation between LL (entries 11–13) and FL (entries 14–16) (see Figure S2), which confirms the interesting flexibility of the process. In addition, the relative composition of the main terpenes seems not to be influenced by the transposition to a larger scale (see Figure 10), meaning that the pilot scale-up not only reached comparable or enhanced extraction yields (see Section 3.2, Table 6), but that it also guarantees the collection of a high-quality product.

**Figure 11:**
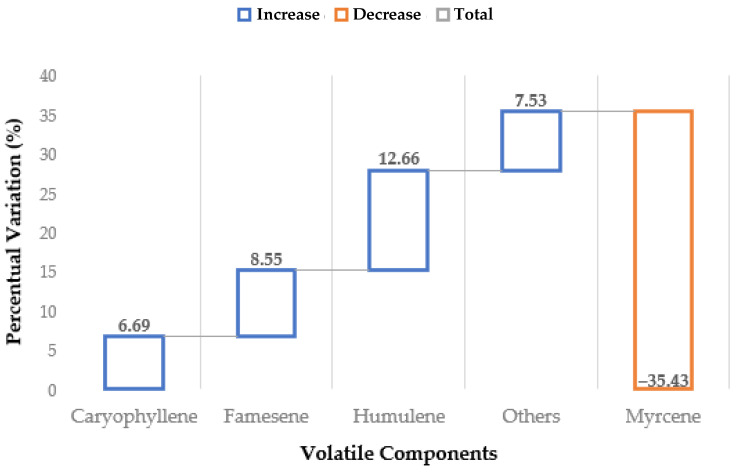
Waterfall chart of main-terpene percentage variation, according to normal and extended MAHD, performed with ETHOS XL.

In order to study the evolution of volatile composition across the pilot protocol, the MAHD carried out on DH was extended by 20 min and the recovered product was also characterized by GC-MS. As shown in Figure 10, it is interesting to note that the relative terpene ratios appear to be unbalanced towards sesquiterpenes in the “extended” process. This is an important point, which indicates that the extraction is not linear during the entire MAHD process. It is plausible that the biomass is almost depleted of myrcene, due to its higher volatility, in the final phase of extraction. Thus, by continuing the distillation, the collected product is progressively enriched with the sesquiterpene fraction. This hypothesis can be supported by a simple evaluation of the percentage variation of terpene yield for the normal and extended protocols. To graphically depict the above reported discussion, it is possible to refer to the waterfall chart in Figure 11 depicting how the accumulation of sesquiterpenes perfectly balances the myrcene contraction, and highlights the progressive enhancement of the first elements. It is reasonable to think that this type of behavior may be exploited to select different product fractions, according to the most desirable flavor. 

## 4. Conclusions

In this study, we have applied MAHD to different types of hops (namely, FH, DH and PH). We first evaluated the variation in total volatile yields provided by several extraction protocols. Considerations as to overall MW irradiation and the soaking approaches were made, and we demonstrated that it is the possible to optimize the extraction procedure according to matrix nature.

After this early-stage screening, the study continued by focusing attention on the scale-up transposition. Lab-scale tests with a 12 L reactor vessel (Section 3.1.3) and a new temperature-detection method paved the way for a pilot reactor (ETHOS XL, Milestone srl, Bergamo, Italy), which is able to process up to 30 kg on each run (Section 3.1.3).

The ETHOS XL reached extraction yields that are comparable with those of the laboratory-scale extractor. This instrument can treat more than 6 times the amount of biomass in a similar period, making this process far more efficient.

A single ETHOS X production run for a whole working day is 40 mL of volatile fraction and a consumption of 6.5 kg of fresh hops. An ETHOS XL in the same working time can produce 144 mL of volatiles and process 32.8 kg of fresh hops. Furthermore, we have verified how the pilot scale can significantly enhance the hydrodistillation yields for DH and PH, compared to small-scale projections; pellet productivity was approximately quadrupled, whilst that of dry cones nearly doubled. This feature is considerably valuable as industry prefers to use dried and pelletized hops as this facilitates storage and preservation.

Finally, the GC-MS analysis evaluated the variation of the four main volatile compounds (Section 3.5). No significant variations were observed, while MAHD protocols were altered. On the other hand, myrcene concentration was considerably influenced by hop preservation, with the highest levels being found in the fresh matrix. Finally, it was observed that the average quantity of sesquiterpenes (caryophyllene, humulene and farnesene) increased, with respect to monoterpenes, upon increasing the extraction time (Section 3.5.3). It is reasonable to assert that this trend may be exploited to select different volatile fractions, according to demand.

## Figures and Tables

**Table 1:** Hops water content.

Hops	Water Content (*w*/*w*, %)	S.D. (*w*/*w*, %)
Fresh	70	2.32
Dried	12	0.41
Pellet	12	0.41

**Table 2:** Mild irradiation MAHD protocol.

Step	Time (min)	Power (Watt)
1	3	500
2	3	1100
3	14	1600
4	90	1500

**Table 3:** MAHD, SOP protocol screening.

Entry	Extraction Material	Hops (g)	L/S (L/kg)	Volatiles (mL)	Yield (mL_VF_/kg)	Dry Yield (mL_VF_/kg_dry matrix_)
1	FH	1200	0.5	6.0	5.00	16.67
2	DH	400	1	3.7	9.25	10.51
3	PHt	1000	3	3.3	3.30	3.75

**Table 4:** MAHD, moistening screening. Extraction material: fresh hops (FH).

Entry	Hops (g)	L/S (L/kg)	Volatiles (mL)	Yield (mL_VF_/kg)	Dry Yield (mL_VF_/kg_dry matrix_)
4	1200	0.25	Burnt biomass, no recovery		
5	1100	1	5.5	5.00	16.67
6	1200	2	5.5	4.58	15.28

**Table 5:** MAHD, mild irradiation protocol and 12 L vessel scale-up. L/S ratio: 1 L/kg.

Entry	Extraction Material	Hops (g)	Volatiles (mL)	Yield (mL_VF_/kg)	Dry Yield (mL_VF_/kg_dry matrix_)
7	FH	1300	8.0	6.15	20.51
8	DH	1000	7.5	7.50	8.52
9	PH	1300	3.2	2.46	2.80
10	FH-Scale-up ^a^	3000	15.9	5.30	17.67

^a^ The extraction protocol was carried out for 360 min.

**Table 6:** MAHD, pilot scale.

Entry	Extraction Material	Hops (g)	Volatiles (mL)	Yield (mL_VF_/kg)	Dry Yield (mL_VF_/kg_dry matrix_)	Label
11	FH ^a^	2500	13.0	5.20	17.33	Low Load
12	DH ^a^	2000	20.7	10.35	11.76	
13	PH ^a^ t	2000	11.0	5.50	6.25	
14	FH ^b^	8200	36.0	4.39	14.63	Full Load
15	DH ^b^	3820	50.0	13.09	14.87	
16	PH ^b^	4000	38.0	9.50	10.80	

^a^ The extraction was carried out for 70 min after reaching 100 °C inside the reactor chamber; ^b^ The extraction was carried out for 120 min after reaching 100 °C inside the reactor chamber.

**Table 7:** Vacuum MAHD. Biomass: Fresh hops (700 g); L/S: 1 L/kg.

Entry	Temperature (°C)	Ramp Irradiation ^a^ (W)	Volatiles (mL)	Yield (mL_VF_/kg)	Dry Yield (mL_VF_/kg_dry matrix_)
17	95	747	1.7	2.43	8.10
18	99	989	1.9	2.71	9.05

^a^ Energy integration of heating step (ramp).

## Supplementary Materials

The following are available online at https://www.mdpi.com/article/10.3390/foods10112726/s1, Table S1: Standard operating procedure (SOP) standardized MAHD protocol, Table S2: ETHOS XL extraction protocols. A: half capacity or lower; B: more than half capacity, Table S3: MAHD vacuum extraction protocol A and B, Table S4: GC temperature protocol, Table S5: MAHD screening summary, Table S6: Energy consumption evaluation. Figure S1: Volatile fraction extraction yields summary, Figure S2: Main terpenoid distribution across MAHD screenings, GC-MS analysis.

## References

[1] Humulus Lupulus. http://www.missouribotanicalgarden.org/PlantFinder/PlantFinderDetails.aspx?kempercode=f191.

[2] International Hop Growers’ Convention—Economic Commission—Summary Reports. http://www.hmelj-giz.si/ihgc/doc/2017APRIHGCECREPORTfinal_LQ_web.pdf..

[3] Holubkova A., Mosovska S., Baloghová B., Šturdík E. (2013). Hop pellets as an interesting source of antioxidant active compounds. Potravinarstvo Slovak J. Food Sci..

[4] Garetz M. (1994). Using Hops: The Complete Guide to Hops for the Craftbrewer.

[5] De Keukeleire D., Verzeli M. (1991). The alpha acids. Chemistry and Analysis of Hop and Beer Bitter Acids.

[6] Katono F., Yonezawa D., Inui T. (2017). Hop Extract And Method For Producing Same. U.S. Patent.

[7] Nance R.M., Setzer W.N. (2011). Volatile components of aroma hops (*Humulus lupulus* L.) commonly used in beer brewing. J. Brew. Distilling.

[8] Kunze W. (1996). Raw materials. Technology Brewing and Malting.

[9] Bocquet L., Sahpaz S., Hilbert J.L., Rambaud C., Rivière C. (2018). Humulus lupulus L., a very popular beer ingredient and medicinal plant: Overview of its phytochemistry, its bioactivity, and its biotechnology. Phytochem. Rev..

[10] Fix G. (1999). Wort Boiling. Principle of Brewing Science.

[11] Peacock V.E., Deinzer M.L. (1981). Chemistry of Hop Aroma in Beer. J. Am. Soc. Brew. Chem..

[12] Formato A., Gallo M., Ianniello D., Montesano D., Naviglio D. (2013). Supercritical fluid extraction of α- and β-acids from hops compared to cyclically pressurized solid–liquid extraction. J. Supercrit. Fluids.

[13] Hrnčič M.K., Španinger E., Košir I.J., Knez Ž., Bren U. (2019). Hop Compounds: Extraction Techniques, Chemical Analyses, Antioxidative, Antimicrobial, and Anticarcinogenic Effects. Nutrients.

[14] Zanoli P., Zavatti M. (2008). Pharmacognostic and pharmacological profile of *Humulus lupulus* L.. J. Ethnopharmacol..

[15] Karabín M., Hudcová T., Jelínek L., Dostálek P. (2016). Biologically Active Compounds from Hops and Prospects for Their Use. Compr. Rev. Food Sci. Food Saf..

[16] Da Silva S.L., Chaar J.D.S., Figueiredo P.D.M.S., Yano T. (2008). Cytotoxic evaluation of essential oil from Casearia sylvestris Sw on human cancer cells and erythrocytes. Acta Amaz..

[17] Sain S., Naoghare P., Devi S., Daiwile A., Krishnamurthi K., Arrigo P., Chakrabarti T. (2014). Beta Caryophyllene and Caryophyllene Oxide, Isolated from Aegle Marmelos, as the Potent Anti-inflammatory Agents against Lymphoma and Neuroblastoma Cells. Antiinflamm. Antiallergy Agents Med. Chem..

[18] Paventi G., De Acutis L., De Cristofaro A., Pistillo M., Germinara G.S., Rotundo G. (2020). Biological Activity of Humulus Lupulus (L.) Essential Oil and Its Main Components Against *Sitophilus granarius* (L.). Biomolecules.

[19] ASBC Methods of analysis. https://www.asbcnet.org/Methods/HopsMethods/Pages/default.aspx.

[20] Burger P., Plainfossé H., Brochet X., Chemat F., Fernandez X. (2019). Extraction of Natural Fragrance Ingredients: History Overview and Future Trends. Chem. Biodivers..

[21] Jeyaratnam N., Abdurahman H.N., Ramesh K., Azhari H.N., Yuvaraj A.R., Akindoyo J.O. (2016). Essential oil from Cinnamomum cassia bark through hydrodistillation and advanced microwave assisted hydrodistillation. Ind. Crop. Prod..

[22] Chemat F., Vian M.A., Cravotto G. (2012). Green Extraction of Natural Products: Concept and Principles. Int. J. Mol. Sci..

[23] Chemat F., Abert-Vian M., Fabiano-Tixier A.-S., Strube J., Uhlenbrock L., Gunjevic V., Cravotto G. (2019). Green extraction of natural products. Origins, current status, and future challenges. Trends Anal. Chem..

[24] Chemat F., Vian M.A., Fabiano-Tixier A.-S., Nutrizio M., Jambrak A.R., Munekata P.E.S., Lorenzo J.M., Barba F.J., Binello A., Cravotto G. (2020). A review of sustainable and intensified techniques for extraction of food and natural products. Green Chem..

[25] Ferreira D.F., Lucas B.N., Voss M., Santos D., Mello P.A., Wagner R., Cravotto G., Barin J.S. (2020). Solvent-free simultaneous extraction of volatile and non-volatile antioxidants from rosemary (*Rosmarinus officinalis* L.) by microwave hydrodiffusion and gravity. Ind. Crop. Prod..

[26] Binello A., Grillo G., Barge A., Allegrini P., Ciceri D., Cravotto G. (2020). A Cross-Flow Ultrasound-Assisted Extraction of Curcuminoids from Curcuma longa L.: Process Design to Avoid Degradation. Foods.

[27] Cheng Y., Xue F., Yu S., Du S., Yang Y. (2021). Subcritical Water Extraction of Natural Products. Molecules.

[28] Tommasi E., Cravotto G., Galletti P., Grillo G., Mazzotti M., Sacchetti G., Samorì C., Tabasso S., Tacchini M., Tagliavini E. (2017). Enhanced and Selective Lipid Extraction from the Microalga P. tricornutum by Dimethyl Carbonate and Supercritical CO2 Using Deep Eutectic Solvents and Microwaves as Pretreatment. ACS Sustain. Chem. Eng..

[29] Samorì C., Mazzei L., Ciurli S., Cravotto G., Grillo G., Guidi E., Pasteris A., Tabasso S., Galletti P. (2019). Urease Inhibitory Potential and Soil Ecotoxicity of Novel “Polyphenols–Deep Eutectic Solvents” Formulations. ACS Sustain. Chem. Eng..

[30] Grillo G., Gunjević V., Radošević K., Redovniković I., Cravotto G. (2020). Deep Eutectic Solvents and Nonconventional Technologies for Blueberry-Peel Extraction: Kinetics, Anthocyanin Stability, and Antiproliferative Activity. Antioxidants.

[31] Rapinel V., Chemat A., Santerre C., Belay J., Hanaei F., Vallet N., Jacques L., Fabiano-Tixier A.-S. (2020). 2-Methyloxolane as a Bio-Based Solvent for Green Extraction of Aromas from Hops (Humulus lupulus L.). Molecules.

[32] Belwal T., Chemat F., Venskutonis P.R., Cravotto G., Jaiswal D.K., Bhatt I.D., Devkota H.P., Luo Z. (2020). Recent advances in scaling-up of non-conventional extraction techniques: Learning from successes and failures. TrAC Trends Anal. Chem..

[33] Grillo G., Boffa L., Binello A., Mantegna S., Cravotto G., Chemat F., Dizhbite T., Lauberte L., Telysheva G. (2019). Cocoa bean shell waste valorisation; extraction from lab to pilot-scale cavitational reactors. Food Res. Int..

[34] Filly A., Fernandez X., Minuti M., Visinoni F., Cravotto G., Chemat F. (2014). Solvent-free microwave extraction of essential oil from aromatic herbs: From laboratory to pilot and industrial scale. Food Chem..

[35] Fidalgo A., Ciriminna R., Carnaroglio D., Tamburino A., Cravotto G., Grillo G., Ilharco L.M., Pagliaro M. (2016). Eco-Friendly Extraction of Pectin and Essential Oils from Orange and Lemon Peels. ACS Sustain. Chem. Eng..

[36] Grillo G., Boffa L., Talarico S., Solarino R., Binello A., Cavaglià G., Bensaid S., Telysheva G., Cravotto G. (2020). Batch and Flow Ultrasound-Assisted Extraction of Grape Stalks: Process Intensification Design up to a Multi-Kilo Scale. Antioxidants.

[37] Derek L. (1978). Hop Extraction with Carbon Dioxide. U.S. Patent.

[38] Forster A., Gehrig M. (1984). Process for The Extraction Of Hop Substances. U.S. Patent.

[39] Panzner F., Evans B.R. (1983). Extraction of Plant Material By Using Carbon Dioxde. U.S. Patent.

[40] Kusuma H., Mahfud M. (2017). Kinetic studies on extraction of essential oil from sandalwood (Santalum album) by microwave air-hydrodistillation method. Alex. Eng. J..

[41] Gunjevic V., Grillo G., Carnaroglio D., Binello A., Barge A., Cravotto G. (2021). Selective recovery of terpenes, polyphenols and cannabinoids from Cannabis sativa L. inflorescences under microwaves. Ind. Crop Prod..

[42] Tyśkiewicz K., Gieysztor R., Konkol M., Szałas J., Rój E. (2018). Essential Oils from Humulus Lupulus scCO2 Extract by Hydrodistillation and Microwave-Assisted Hydrodistillation. Molecules.

[43] Markle S. (2019). Strain-Specific Isolation of Terpenes Utilizing Microwave-Assisted Extraction. Cannabis Sci. Technol..

[44] Ciriminna R., Fidalgo A., Delisi R., Carnaroglio D., Grillo G., Cravotto G., Tamburino A., Ilharco L.M., Pagliaro M. (2017). High-Quality Essential Oils Extracted by an Eco-Friendly Process from Different Citrus Fruits and Fruit Regions. ACS Sustain. Chem. Eng..

