# Supplementary material for: Microwave-Assisted Hydrodistillation of Hop (Humulus lupulus L.) Terpenes: A Pilot-Scale Study
Source: Foods. 2021 Nov 7;10(11):2726. doi: 10.3390/foods10112726 (PMC8625699; doi:10.3390/foods10112726)
Supplement: Supplementary file 1 [file foods-10-02726-s001.zip › foods-1413636-supplementary.pdf]

Supporting Information

Microwave-Assisted Hydrodistillation of Hop (*Humulus lupulus* L.) Terpenes: A Pilot-Scale Study

Lorenzo Lamberti[1,2], Giorgio Grillo[1], Lorenzo Gallina[1], Diego Carnaroglio[3], Farid Chemat[4] and Giancarlo Cravotto[1,*]

[1] Dipartimento di Scienza e Tecnologia del Farmaco, University of Turin, Via P. Giuria 9, 10125, Turin, Italy;
[2] Baladin S.S.Agricola, via Carrù 23, 12060 Piozzo, Cuneo, Italy;
[3]Milestone srl, via Fatebenefratelli, 1-5, Sorisole 24010, Italy.
[4] Avignon University, INRAE, UMR 408, GREEN Extraction Team, F-84000 Avignon, France.
* Correspondence: Correspondence: giancarlo.cravotto@unito.it;

Entries reported in the document (Table S5 and S6, Figure S1 and S2) refers to the same samples.

Table S1. Standard Operating Procedure (SOP) standardized MAHD protocol.

Step	Time (min)	Power (W)
1	15	1800
2	75	1100

Table S2. ETHOS XL extraction protocols. A: half capacity or lower; B: more than half the capacity.

A: Low Loading (LL)

Step	Time (min)	Power (W)
1	Up to 100°C	4000
2	10	4000
3	60	3200

B: Full Loading (FL)

Step	Time (min)	Power (W)
1	Up to 100°C	4000
2	30	4000
3	90	3200

Table S3. MAHD vacuum extraction protocol A and B.

Protocol A

Step	Time (min)	Power (W)	Temperature (°C)
1	10	1200	95
2	100	1200	95

Protocol B

Step	Time (min)	Power (W)	Temperature (°C)
1	10	1800	99
2	100	1600	99

Table S4. GC temperature protocol.

°C/min.	Final temperature	Hold
0	35	2
5	65	0
2	85	0
10	130	0
2	150	0
10	300	0

Table S5. MAHD screening summary.

Entry	Hops Type	Hops Weight (g)	L/S (L/kg)	Volatiles (mL)	Yield (ml$_{VF}$/kg)	Dry Yield (mL$_{VF}$/kg$_{dry\ matrix}$)	Type of evaluation
1	FH	1200	0.5	6	5.00	16.67	SOP
2	DH	400	1	3.7	9.25	10.51	
3	PH	1000	3	3.3	3.30	3.75	
4	FH	1200	0.25	*Burned material, no recovery*			Moistening evaluation
5	FH	1100	1	5.5	5.00	16.67	
6	FH	1200	2	5.5	4.58	15.28	
7	FH	1300	1	8.0	6.15	20.51	Mild MAHD
8	DH	1000	1	7.5	7.50	8.52	
9	PH	1300	1	3.2	2.46	2.80	
10	FH	3000	1	15.9	5.30	17.67	
11	FH	2500	0.5	13.0	5.20	17.33	Pilot (LL)
12	DH	2000	1	20.7	10.35	11.76	
13	PH	2000	1	11.0	5.50	6.25	
14	FH	8200	0.5	36.0	4.39	14.63	Pilot (FL)
15	DH	3820	1	50.0	13.09	14.87	
16	PH	4000	1	38.0	9.50	10.80	
17	FH	700	1	1.7	2.43	8.10	Vacuum MAHD
18	FH	700	1	1.9	2.71	9.05	

FH: Fresh hops; DH: Dried hops; PH: Pelletized hops.

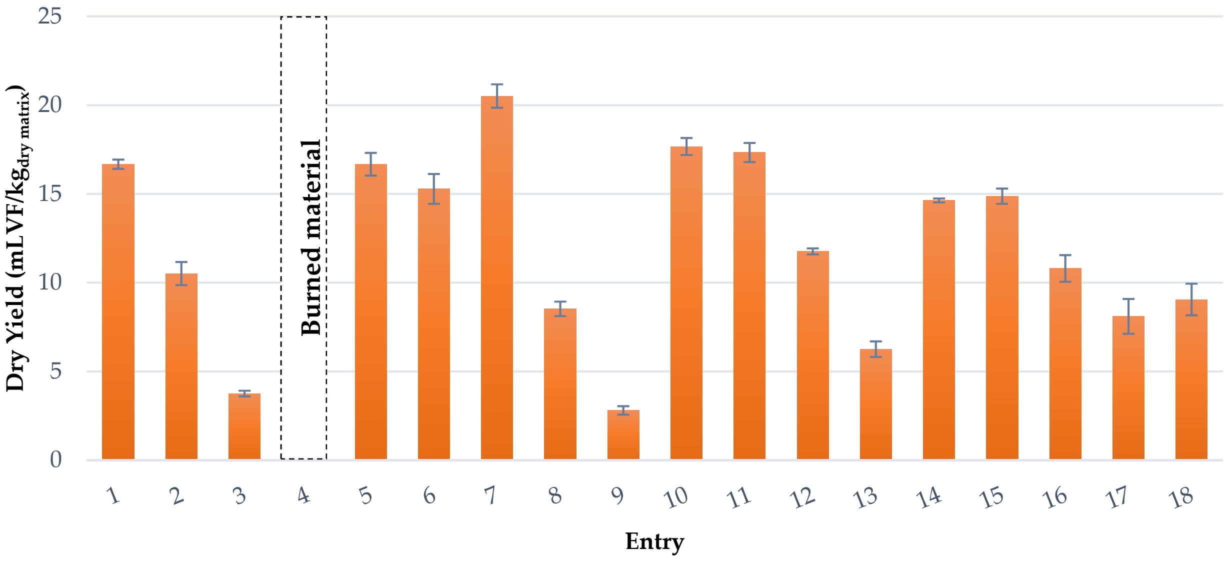

Figure S1. Volatiles fraction extraction yields summary. Results reported as average ± SD.

Table S6. Energy consumption evaluation.

Entry	Volatile Fraction (mL)	Energy Consumption	
		(kJ)	(kJ/mL$_{VF}$)
1	6	22770	3795
2	3.7	22770	6154
3	3.3	22770	6900
4	-	22770	-
5	5.5	22770	4140
6	5.5	22770	4140
7	8	29532	3692
8	7.5	29532	3938
9	3.2	29532	9229
10	15.9	97032	6103
11	13	40320	3102
12	10.5	40320	3840
13	11	40320	3665
14	36	65880	1830
15	50	65880	1318
16	38	65880	1734
17	1.7	22321	13130
18	1.9	24466	12877

Note: In the energy consumption evaluation, both reactors and respective chiller has been taken in account.

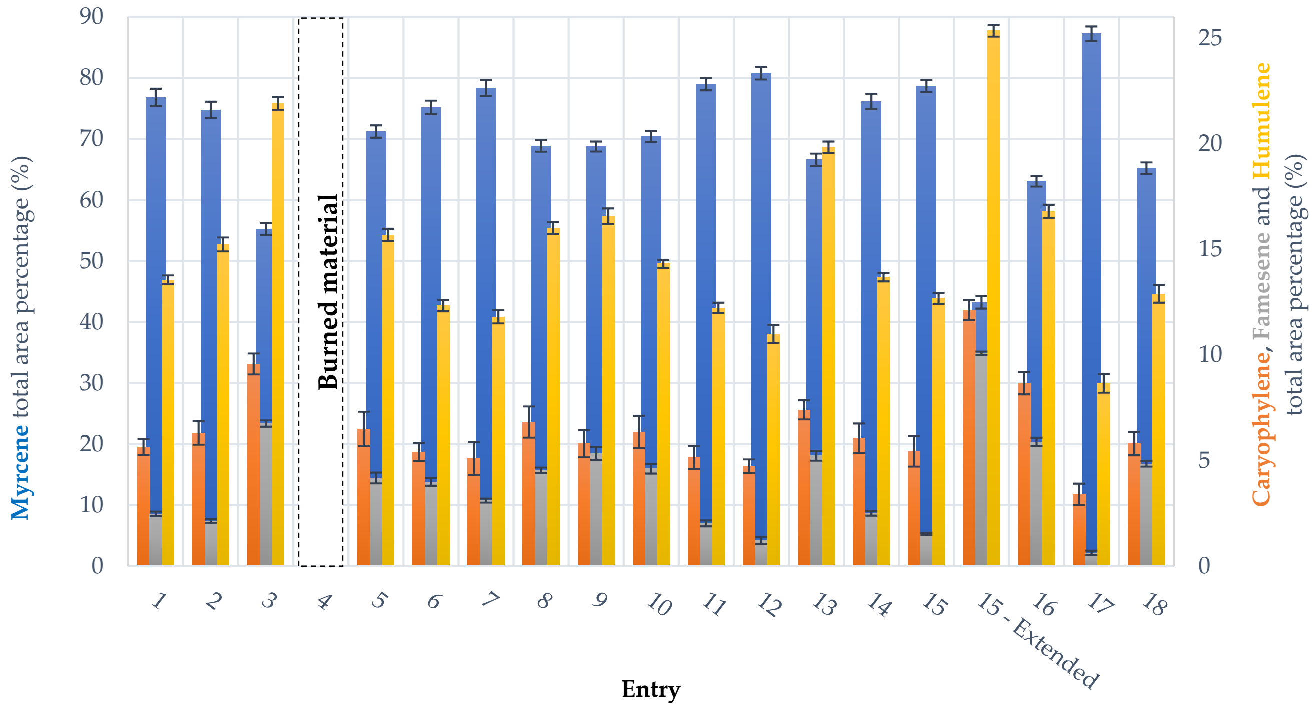

Figure S2. Main terpenoids distribution across MAHD screening, GC-MS analysis. Entry 15 – extended are reported in Paragraph 3.2.3. Results reported as average ± SD.
